# Effects of a glucosinolate‐rich beverage on blood lactate concentrations during submaximal exercise

**DOI:** 10.14814/phy2.71081

**Published:** 2026-08-25

**Authors:** Fredrik Vinge, Emma Tillqvist, Oscar Horwath, William Apró, Filip J. Larsen, Michaela L. Sundqvist

**Affiliations:** ^1^ Department of Physiology, Nutrition and Biomechanics The Swedish School of Sport and Health Sciences Stockholm Sweden; ^2^ Department of Physiology and Pharmacology Karolinska Institute Stockholm Sweden; ^3^ Department of Women's and Children's Health Karolinska Institute Stockholm Sweden

**Keywords:** blood lactate, glucosinolates, submaximal exercise

## Abstract

Glucosinolate‐rich red kale sprouts (GRS) combined with intense exercise training for 7 days reduce blood lactate during exercise, attenuate hypoglycemic events, improve performance, and reduce markers of oxidative stress. This study aimed to investigate the acute, dose‐dependent effects of GRS on blood lactate and blood glucose after ingestion of two different doses. In the study, healthy participants (*n* = 15) consumed 37.5 or 75 g of GRS or an isocaloric placebo blended into a beverage on three occasions. They cycled on an ergometer at three submaximal work rates (~100, ~120, ~140 W, respectively) before and 3 h after ingestion of GRS. Oxygen uptake, substrate‐level oxidation, blood lactate, blood glucose, and ratings of perceived exertion were taken before and after each interval. GRS intake acutely decreased blood lactate during submaximal cycling and increased resting blood glucose. The largest lactate reduction occurred at 37.5 g compared to placebo, with concentrations 0.4 ± 0.6 mM lower at the highest work rate (*p* = 0.00175). For 75 g, the reduction was 0.3 ± 0.5 mM (*p* = 0.0484). No significant effects appeared at the lowest work rate. Resting blood glucose averaged 3.9 ± 0.3 mM after placebo compared to 4.3 ± 0.4 mM after 37.5 g and 4.3 ± 0.4 mM after 75 g GRS (*p* < 0.0001, respectively). In conclusion, these findings suggest that acute intake of a glucosinolate‐rich beverage may influence lactate and glucose metabolism, with lower blood lactate concentrations observed at two submaximal work rates and altered blood glucose concentrations at rest.

## 1 INTRODUCTION

In endurance exercise, lactate accumulation is closely correlated with endurance performance (Sjödin & Svedenhag, 1985). During incremental exercise, the second lactate threshold (LT2) is characterized by a non‐linear increase in blood lactate concentration, reflecting a transition toward an increased metabolic strain. This threshold does not correspond to a fixed blood lactate concentration but shows considerable inter‐individual variability and can be altered by exercise training (Faude et al., 2009). Consequently, lower lactate levels at submaximal exercise intensities are a hallmark of positive adaptations to a period of aerobic endurance exercise training (Hurley et al., 1984). More recently, regular lactate measurements have also gained a central role in advancing training models, such as lactate‐guided threshold interval training (LGTIT). This approach tailors training intensity based on an athlete's blood lactate levels and has shown beneficial results when implemented in elite runners (Casado et al., 2023).

To lower lactate levels during exercise, different pharmacological approaches have also been explored. One recognized attempt was the use of dichloroacetate (DCA), a drug that stimulates pyruvate dehydrogenase activity. Activation of pyruvate dehydrogenase by DCA promotes the utilization of pyruvate in the mitochondria, thereby decreasing lactate accumulation. While DCA consistently reduces lactate levels during submaximal cycling, its effectiveness in improving exercise performance seems to be context‐dependent (Calvert et al., 2008; Carraro et al., 1989; Timmons et al., 1998).

Recently, a specific group of phytochemicals, isothiocyanates, has gained attention for their ability to alter metabolic responses and gene expression during exercise. Isothiocyanates, naturally found in cruciferous vegetables like kale, broccoli, and cabbage, are derived from the hydrolysis of glucosinolates, a reaction catalyzed by the enzyme myrosinase (Fahey et al., 1997). Supplementation with the most well‐studied isothiocyanate, sulforaphane (SFN), has been shown to activate the transcription factor Nuclear Factor Erythroid 2‐Related Factor 2 (Nrf2) (Fahey et al., 1997). Nrf2 is recognized as the primary regulator of cellular oxidative stress and has been identified as a key coordinator of the adaptive response to exercise training (Vargas‐Mendoza et al., 2019). In a study by Oh et al. (2017), mice receiving intraperitoneal injections of SFN for 3 days prior to exhaustive exercise exhibited reduced oxidative stress markers and improved running capacity. Also, treatment with SFN in animal exercise studies under hypoxic conditions resulted in lower plasma lactate levels, increased expression of monocarboxylate transporters (MCTs), and enhanced lactate dehydrogenase (LDH) activity, which may facilitate the conversion of lactate to pyruvate under certain metabolic conditions (Wang et al., 2020). Furthermore, SFN treatment during a 6‐week high‐intensity interval training (HIIT) protocol in mice improved the antioxidative capacity of skeletal muscle, in response to acute exhaustive exercise, by increasing protein expression of Nrf2 (Wang et al., 2022).

Our research group recently conducted a human trial in which participants consumed 75 g of glucosinolate‐rich broccoli sprouts twice daily for 7 consecutive days, during a period of high‐intensity interval training (HIIT). The results showed a reduction in oxidative stress markers, decreased lactate levels during submaximal cycling, and enhanced physical performance compared to the placebo treatment (Flockhart et al., 2023). This single‐blinded, randomized, placebo‐controlled crossover study aimed to assess the acute effects of glucosinolate‐rich red kale sprouts (GRS) on lactate and glucose metabolism during submaximal cycling, 3 h after consuming varying doses (37.5 or 75 g) of GRS or a placebo beverage based on alfalfa sprouts (PLA).

## 2 METHOD

### 2.1 Ethical approval

The study was conducted in accordance with the principles of the Declaration of Helsinki (2024), except for registration in a public database. Ethical approval was obtained from the Swedish Ethical Review Authority (approval no. 2019‐02194). All participants were informed about the study aims, procedures, and potential risks prior to participation and provided written informed consent before inclusion in the study.

### 2.2 Study design

The study employed a single‐blind, randomized, crossover design. Researchers were aware of the beverage allocation, while participants were blinded. Each participant completed three visits, separated by 1 week, with beverages administered in a counterbalanced order. At each visit, participants first cycled at three predetermined submaximal intensities, consumed the assigned beverage, and then rested in a fasted state for 3 h before repeating the same cycling protocol.

### 2.3 Study population

Participants were recruited through advertisements at the Swedish School of Sports and Health Sciences (GIH) and via a study‐participant recruitment website (Accindi.com). Participants were eligible if they were males or females aged 18–40 years, representing all levels of physical activity and without any severe chronic disease such as liver disease, heart disease, or diabetes. Written informed consent was obtained from all participants prior to their inclusion in the study. A total of 23 participants were screened, and 11 females and 4 men completed the study. One participant was unable to complete cycling on the ergometer during the screening, was excluded, and those who chose to discontinue the study (*n* = 7) were classified as dropouts. Baseline characteristics are presented in Table 1.

**TABLE 1:** Baseline characteristics, *n* = 15 participants, mean ± SD.

	Female	Male	Total
*n*	11	4	15
Age (years)	27.6 ± 5.4	25.0 ± 2.2	26.9 ± 4.8
Body weight (kg)	63.9 ± 6.5	76.9 ± 3.1	67.4 ± 8.2
Height (cm)	167.3 ± 2.7	177.8 ± 3.2	170.1 ± 5.5
VO_2_max (mL/min^−1^ kg^−1^)	38.5 ± 5.5	47.8 ± 6.6	40.9 ± 7.0

### 2.4 Standardization of food intake and training

Participants were advised to maintain their normal diet but avoid consuming cruciferous vegetables (e.g., broccoli, kale) and nitrate‐rich vegetables (e.g., lettuce, beetroot) 3 days before the laboratory visits. Participants avoided nitrate‐rich foods to standardize baseline nitrate/nitrite levels and isolate intervention effects. This also minimizes potential interactions between isothiocyanates and nitrate. They were also instructed to keep a 24‐h dietary record the day before the first visit, and this recorded food intake was replicated for the subsequent test days to ensure standardized dietary intake. Participants were further instructed to refrain from caffeine intake on test days and to avoid any exercise training 36 h before visits.

### 2.5 Quantification of glucosinolates and isothiocyanates

Glucosinolate measurement was performed following the method of Mawlong et al. (2017), with minor modifications. Freeze‐dried sprouts were homogenized in 80% methanol and then centrifuged at 3000 rpm for 4 min at room temperature, and the supernatant was collected. A 50 μL aliquot was used for analysis, mixed with 50 μL methanol and 250 μL of 2 mM STCP solution (prepared by dissolving 58.85 mg sodium tetrachloropalladate in 170 μL concentrated HCl and 100 mL dH_2_O). The mixture was thoroughly mixed and immediately analyzed via spectrophotometry at 425 nm. Sinigrin was used as the internal standard.

L‐sulforaphane was measured at the Sweden Metabolomics Center in Umeå with Liquid Chromatography‐Mass Spectrometry (LC–MS) in four sprout varieties from cruciferous vegetables, including broccoli sprouts and red kale sprouts. Sample preparation was performed according to (Trygg et al., 2005) with some modifications. Before LC–MS analysis, samples were re‐suspended in methanol and water. Analysis was performed in both positive and negative ion modes using an Agilent 1290 Infinity UHPLC system (Agilent Technologies, Waldbronn, Germany) coupled with an Agilent 6546 Q‐TOF mass spectrometer. Chromatographic separation was achieved on a C18 column with gradient elution. Reference ions were used for accurate mass calibration. Instrument parameters, including gas temperature, flow rates, and voltages, were optimized for detection. Data processing was conducted using Agilent MassHunter Profinder (Agilent Technologies Inc., Santa Clara, USA), with targeted feature extraction for isothiocyanate metabolites. Concentrations of isothiocyanates were determined based on calibration curves. A detailed description of the methods can be found in the supplemental material.

### 2.6 Preparation of red kale sprout beverage

Red kale sprouts were selected for the intervention as they provided the highest concentration of total glucosinolates and sulforaphane. Each bottle contained sprout concentrate equivalent to 37.5 g of fresh sprouts and in the GRS bottles this was equivalent to 606 mg glucosinolates and 8 mg of sulforaphane, 13 g carbohydrates, and ~62 kcal. Accordingly, 75 g fresh sprouts contained 1212 mg glucosinolates, 16 mg sulforaphane, 26 g carbohydrates, and ~ 124 kcal. The placebo beverage based on alfalfa sprouts contained negligible amounts of glucosinolates and 13 g of carbohydrates and ~62 kcal. The doses administered in this study were fixed absolute quantities (37.5 g and 75 g) and were standardized across participants rather than adjusted for body mass, as the bioavailability of GRS not necessarily is expected to scale linearly with body mass. Also, relative dosing based on body mass was not feasible because all beverages were prepared prior to study initiation, making individualized adjustments impossible. Larger doses of GRS than 75 g were tested in a pilot study but not well tolerated by participants and were therefore excluded from the final protocol.

To produce the study beverages the GRS and PLA were pressed, strained, and mixed with water and black currant cordial. Subsequently, the beverage underwent low‐temperature pasteurization and was bottled in 250 mL glass bottles. The GRS and PLA beverages used in the study were produced by Balsgård FoodTech, Sweden, an external contract manufacturer responsible for all production steps, including the preparation and bottling of the beverages. Upon delivery in the laboratory, the entire batch was frozen immediately and thawed 30 min before use, ensuring consistent taste and glucosinolate concentration throughout the study period. To enhance the conversion of potentially remaining glucosinolates to isothiocyanates, 0.75 g of myrosinase‐rich brown mustard seeds were mixed into the drink before consumption.

### 2.7 Pre‐tests

During the screening visit, blood pressure (BP) measurements were taken in the left arm after 5 min of rest in a supine position. Three measurements, with 60 s between each, were performed using an automatic blood pressure monitor (M2, Omron, Kyoto, Japan). The average of the two last measurements was considered the subject's BP. Following these measurements, participants were familiarized with cycling on the cycle ergometer (PT1, Wattbike, Nottingham, UK), and their lactate concentrations were determined at five different submaximal intensities. Exercise intensities were individualized based on an interview regarding each participant's training history and selected to target a blood lactate concentration range of 2–6 mM. The work rate was increased in increments of 10–20 W at each intensity. Between the 5 min long cycling intervals, participants rested for 1 min, during which one capillary blood sample was drawn from the fingertip, then analyzed for glucose and lactate using a Biosen C‐Line analyzer (EKF Diagnostics, Cardiff, UK). According to the manufacturer (EKF‐Diagnostics), the analyzer has a reported coefficient of variation of < 3% for both lactate and glucose. The submaximal cycling test was succeeded by an incremental VO_2_max test to determine maximal oxygen uptake. A facemask with a turbine was attached to participants during both submaximal‐ and maximal cycling to analyze oxygen consumption (Quark CPET, Cosmed Albano Laziale, Italy) and a heart rate monitor (Polar Electro OY, Kempele, Finland) was attached to the chest during cycling.

### 2.8 Dose–response test

On test days, participants arrived in the morning on three independent days, separated by 7 days. The exercise tests were conducted at the same time of day for all tests. Body weight and blood pressure measurements, along with resting capillary blood glucose and lactate, were taken. This was followed by cycling at three submaximal intensities. The resistance on each interval was based on the screening threshold test and aimed to reach 2, 3, and 4 mM lactate after 5 min cycling. The mean effect (Watt) for the three different submaximal work rates on the ergometer bike (participants *n* = 15) was 104 ± 44 W, 124 ± 46 W, and 140 ± 49 W, respectively. After completion of the three intervals, participants ingested a beverage containing either 0 g GRS (PLA), 37.5 g GRS (~8 mg SFN), or 75 g GRS (~16 mg SFN) together with 0.75 g of mustard seeds. Three hours after beverage intake, participants repeated the cycling protocol, followed by blood sampling. This timing was based on previous findings indicating that plasma isothiocyanate concentrations peak approximately 3 h after supplementation, declining significantly by 6 h and returning to near baseline within 24 h (Orlando et al., 2022). Measurement of plasma isothiocyanate concentrations would have required multiple additional venous blood samples; therefore, this was not included in the study protocol.

Myeloperoxidase was measured in EDTA‐plasma using the Human Myeloperoxidase ELISA Kit according to the manufacturer's instructions (Abcam, ab272101, Cambridge, UK). Between the pre‐ and post‐test, participants were fasting but allowed to drink water ad libitum.

### 2.9 Statistical analysis

A linear mixed model was used to analyze the dose‐dependent effects on the change in blood lactate and glucose from pre to post as response variables. The analysis was conducted using Python, with the Statsmodels library for statistical modeling. Comparison between doses was made using the placebo dose as the reference category. This categorization facilitated the comparison of treatment effects across different dosage levels. The model accounted for fixed effects of the dose, VO2max, and baseline levels of lactate and glucose, both at rest and under submaximal exercise. Additionally, each subject was treated as a random effect to account for the inter‐subject variability. This approach allowed us to assess the effect of the treatment while controlling for individual differences and baseline metabolic states. A *p*‐value below 0.05 was considered significant, and data are presented as mean ± SD.

## 3 RESULTS

### 3.1 A glucosinolate‐rich beverage lowers blood lactate during submaximal cycling

**FIGURE 1:**
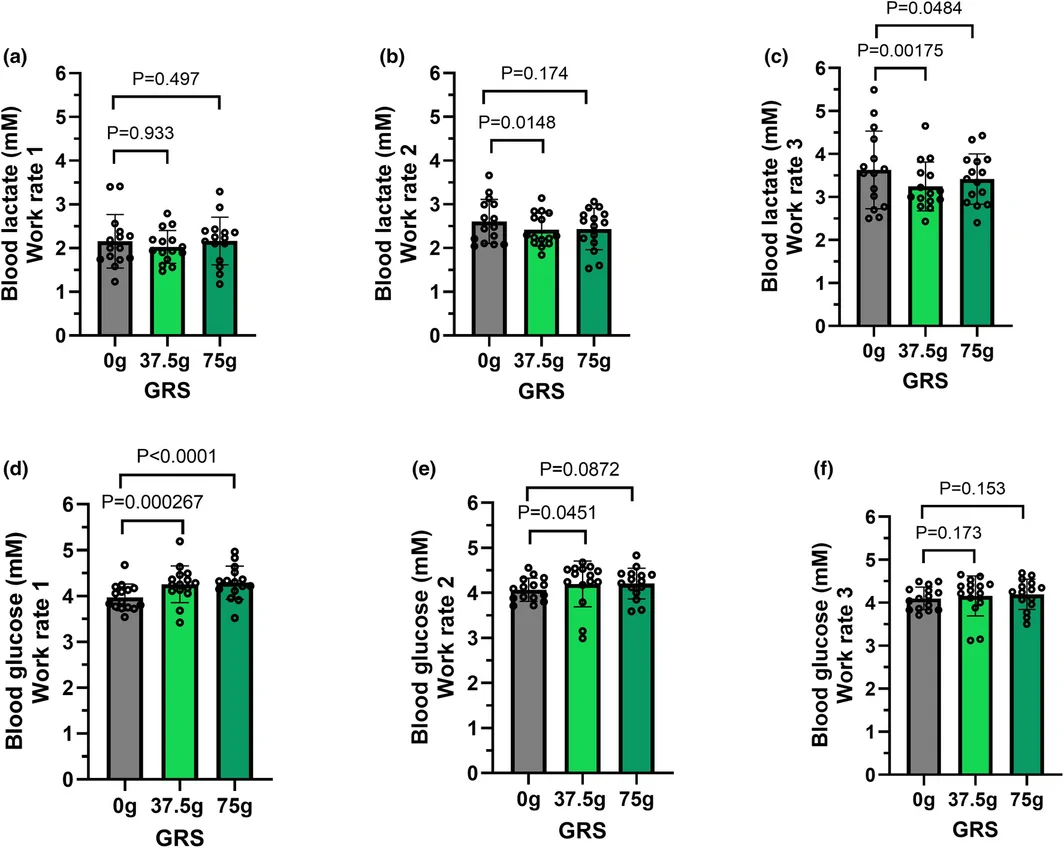
Capillary blood lactate levels (a–c) and capillary blood glucose levels (d–f) during cycling at three work rates on an ergometer bike, measured 3 h after intake of a beverage containing 0, 37.5, or 75 g GRS. Data are presented as mean ± SD. Each dot represents one participant at the respective time point (*n* = 15). *p* < 0.05 is considered significant.

**FIGURE 2:**
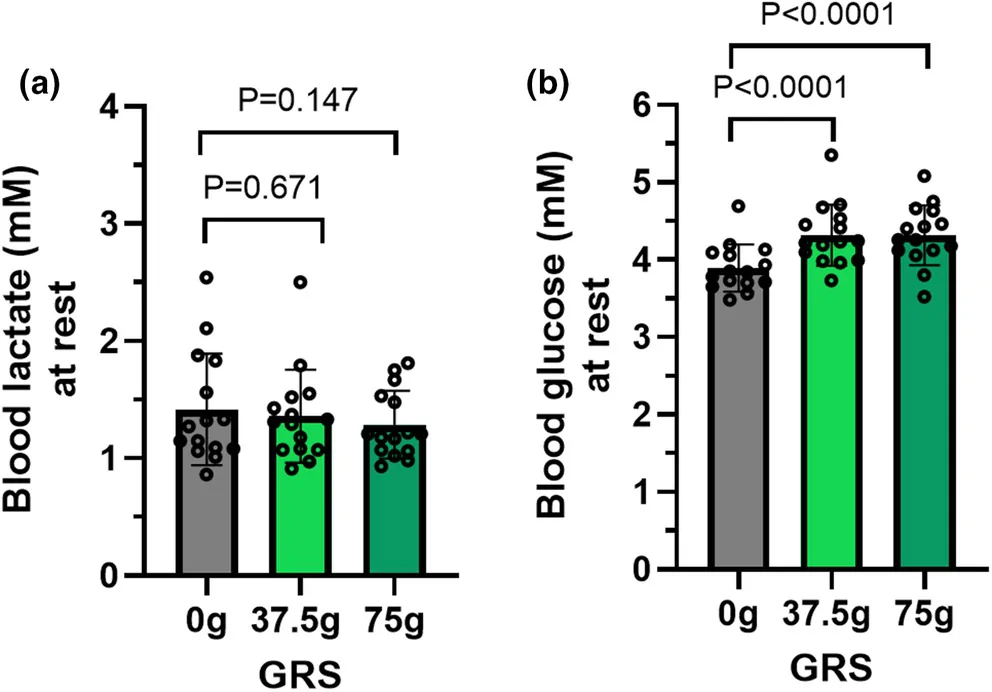
Capillary blood lactate (a) and blood glucose (b) levels during rest, measured 3 h after intake of a beverage containing 0, 37.5, or 75 g of GRS. Data are presented as mean ± SD. Each dot represents one participant at the respective time point (*n* = 15). *p* < 0.05 is considered significant.

Consumption of GRS reduced the blood lactate concentration in study participants (*n* = 15) at a given absolute work rate compared to PLA. The largest reduction was observed at the 37.5 g dose indicating a U‐shaped dose–response relationship. Compared to PLA the lactate concentration was 0.2 ± 0.3 mM lower at work rate 2 after 37.5 g GRS (2.42 mM ± 0.38 vs. 2.61 mM ± 0.50; *p* = 0.0148) and 0.4 ± 0.6 mM lower at work rate 3 (3.25 mM ± 0.57 vs. 3.63 mM ± 0.90; *p* = 0.00175; Figure 1). For the 75‐gram dose, the reduction was 0.1 ± 0.3 mM at work rate 2 (2.43 mM ± 0.47 vs. 2.61 mM ± 0.50; *p* = 0.174) and 0.3 ± 0.5 mM at work rate 3 (3.42 mM ± 0.58 vs. 3.63 mM ± 0.90; *p* = 0.0484). No significant effects were seen in the lowest work rate, where the mean lactate concentration was 2.16 mM ± 0.61 for PLA, 2.03 mM ± 0.38 for 37.5 g GRS and 2.16 mM ± 0.55 for 75 g GRS (*p* = 0.933 and *p* = 0.497, respectively). Resting lactate in the morning compared to resting lactate after a 3‐h fasting rest were not altered (PLA: 1.42 mM ± 0.47; 37.5 g GRS: 1.36 mM ± 0.40; 75 g GRS: 1.29 mM ± 0.29; *p* = 0.671 and *p* = 0.147, respectively; Figure 2). At the individual level, blood lactate concentrations were lower than PLA in 8 of 15 and 10 of 15 participants at work rate 2 following 37.5 g and 75 g GRS, respectively. Corresponding reductions at work rate 3 were observed in 10 of 15 and 12 of 15 participants, respectively.

### 3.2 Blood glucose was normalized after ingesting a glucosinolate‐rich beverage

During submaximal cycling, blood glucose after 37.5 g GRS was significantly higher both at work rate 1 (*p* = 0.000267) and work rate 2 (*p* = 0.0451) compared to PLA, but not at work rate 3 (*n* = 15 participants; *p* = 0.173; Figure 1). Similarly, blood glucose after 75 g GRS was significantly higher at work rate 1 (*p* < 0.0001), but not at work rate 2 (*p* = 0.0872) or work rate 3 (*p* = 0.153) compared to PLA. Resting blood glucose after ingesting 37.5 g and 75 g GRS was significantly higher compared to PLA intake (*p* < 0.0001, respectively; Figure 2). Specifically, after 3 h of rest, the mean glucose concentration was 3.9 ± 0.3 mmol/L following PLA, compared to 4.3 ± 0.4 mM after 37.5 g and 4.3 ± 0.4 mM after 75 g GRS. In total, 10 out of 15 participants were hypoglycemic (below 4 mM blood glucose) after PLA compared to 4 participants when supplementing with 37.5 g GRS and 2 participants when supplementing with 75 g.

### 3.3 A glucosinolate‐rich beverage did not affect blood pressure or heart rate

**FIGURE 3:**
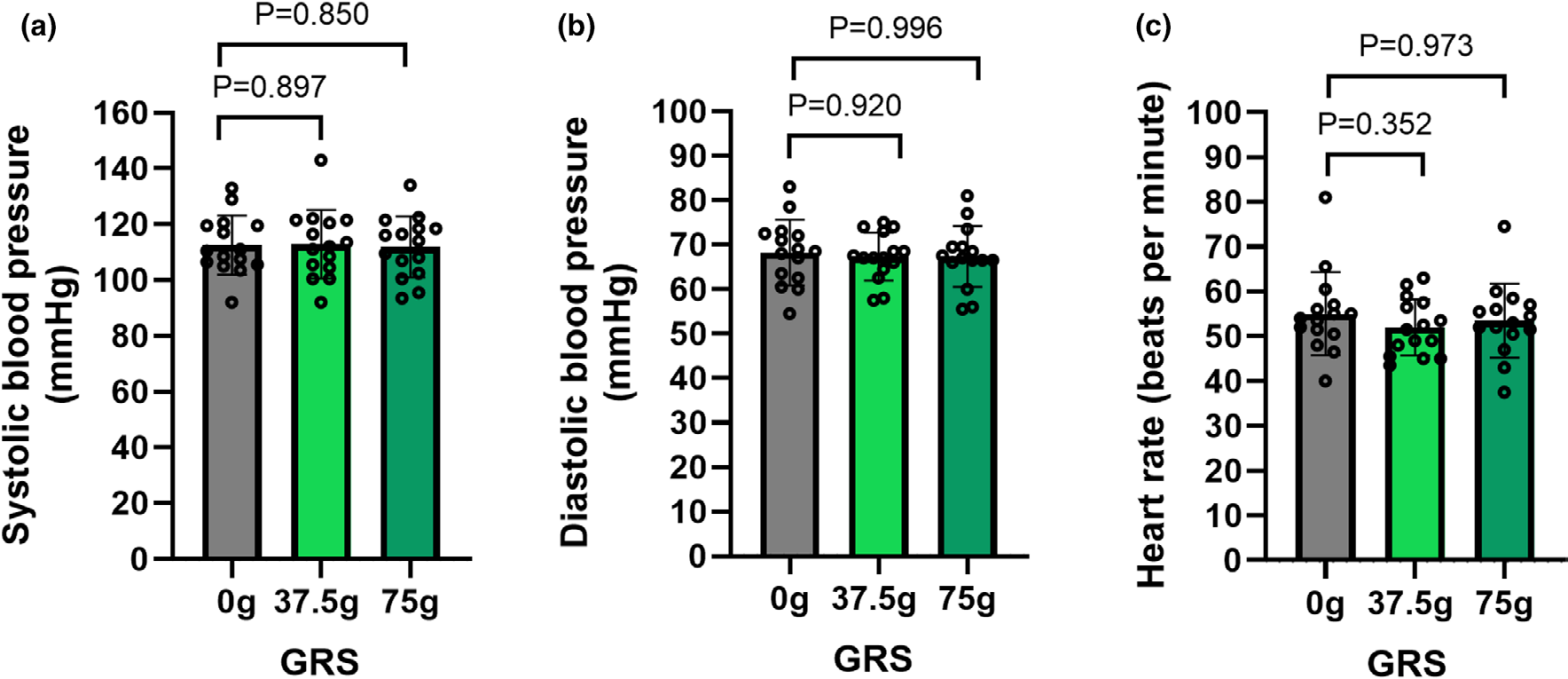
Resting systolic blood pressure (a), diastolic blood pressure (b), and heart rate (c), measured 3 h after intake of a beverage containing 0, 37.5, or 75 g of GRS. Data are presented as mean ± SD. Each dot represents one participant at the respective time point (*n* = 15). *p* < 0.05 is considered significant.

There was no difference in resting systolic BP (37.5 g GRS: *p* = 0.897, 75 g GRS: *p* = 0.850) or diastolic BP (37.5 g GRS: *p* = 0.920, 75 g GRS: *p* = 0.996) or HR (37.5 g GRS: *p* = 0.352, 75 g GRS: *p* = 0.973) among study participants (*n* = 15) after intake of GRS compared to PLA (Figure 3).

### 3.4 Myeloperoxidase

**FIGURE 4:**
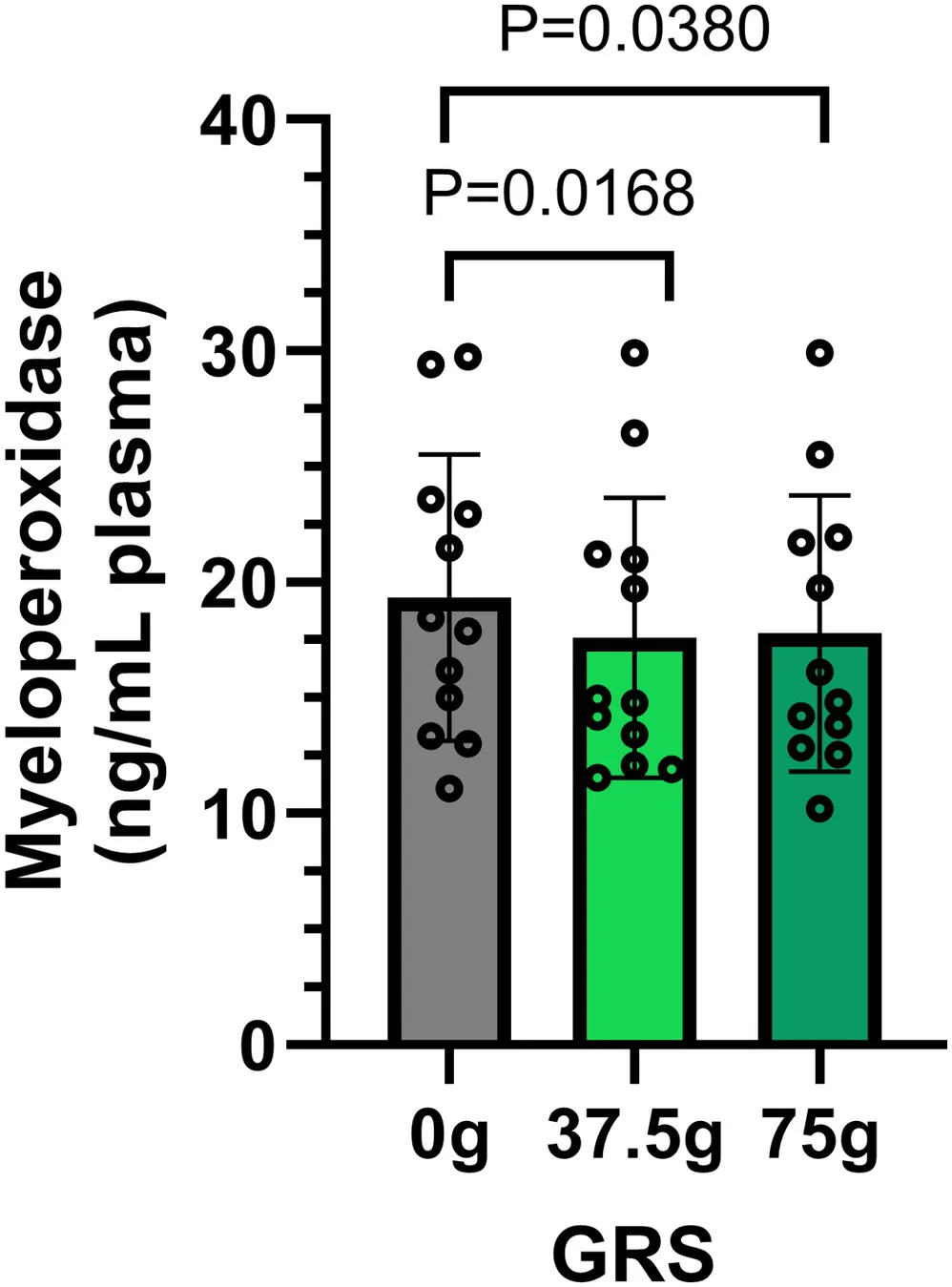
Mean myeloperoxidase levels 3 h after intake of a beverage containing 0, 37.5, or 75 g GRS. Data are presented as mean ± SD. Each dot represents one participant at the respective time point (*n* = 15). *p* < 0.05 is considered significant.

Consistent with our previous findings, which showed reduced MPO levels after 1 week of GRS supplementation (Flockhart et al., 2023), the acute intake of GRS also resulted in lower MPO concentrations. Mean myeloperoxidase levels were lower after 37.5 g and 75 g GRS (*n* = 15 participants; 17.6 ± 6.1 ng/mL, *p* = 0.0168 and 17.8 ± 6.0 ng/mL, *p* = 0.0380, respectively), compared to PLA (19.3 ± 6.1 ng/mL; Figure 4).

### 3.5 Oxygen uptake, substrate use, and ratings of perceived exertion and heart rate

**FIGURE 5:**
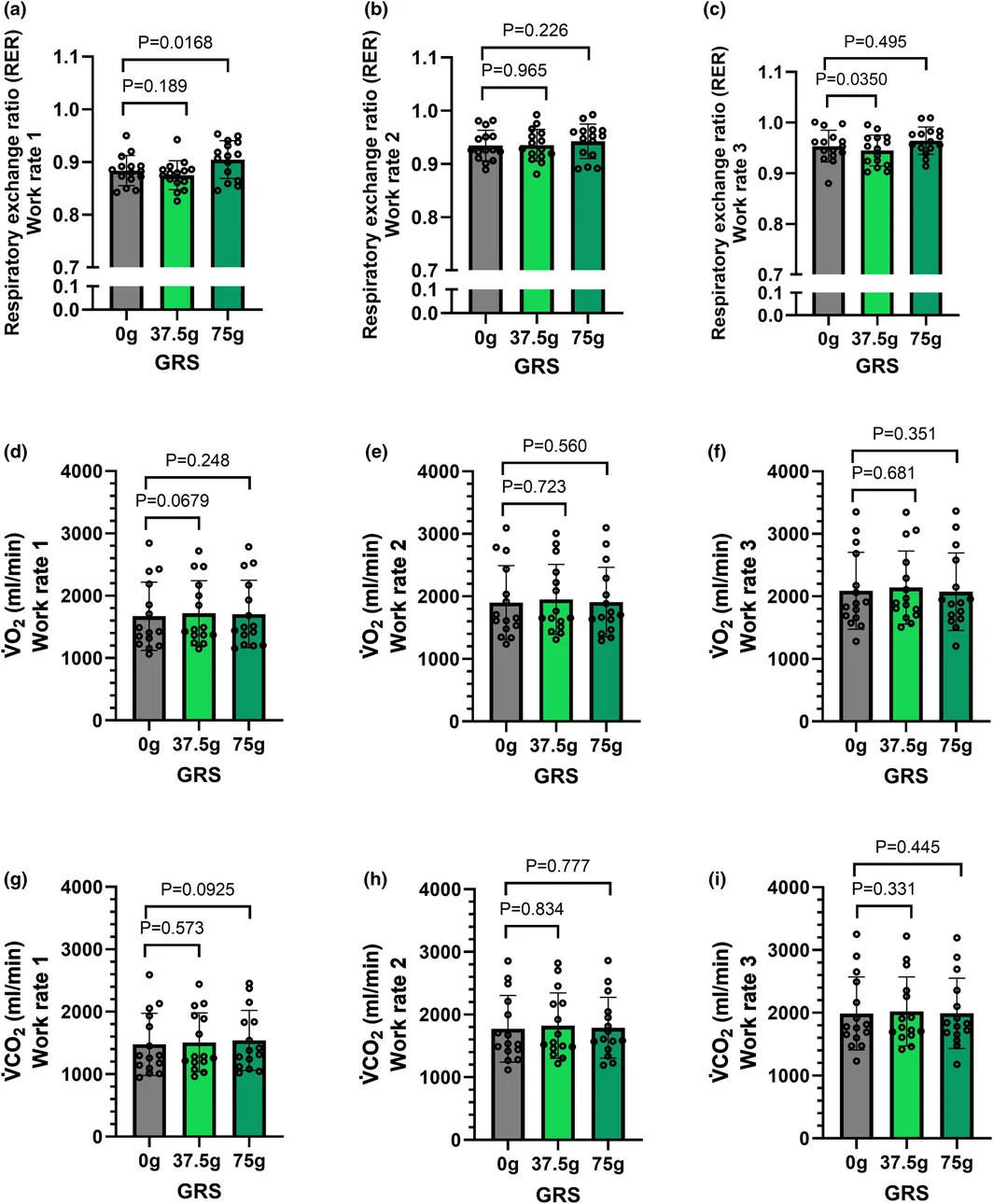
Respiratory exchange ratio (a–c), volume O_2_ consumed (d–f), and CO_2_ produced (g–i) during cycling at three work rates on an ergometer bike, measured 3 h after intake of a beverage containing 0, 37.5, or 75 g GRS. Data are presented as mean ± SD. Each dot represents one participant at the respective time point (*n* = 15). *p* < 0.05 is considered significant.

During work rate 1, the respiratory exchange ratio (RER) in the study participants (*n* = 15) increased following the intake of 75 g GRS (*p* = 0.0168), but not 37.5 g GRS (*p* = 0.189, Figure 5). At work rate 2, no significant differences were observed between conditions (37.5 g GRS: *p* = 0.965; 75 g GRS: *p* = 0.226). At work rate 3, RER was significantly reduced following the intake of 37.5 g GRS (*p* = 0.0350), but not 75 g GRS (*p* = 0.495). No differences in oxygen consumption compared to PLA in participants (*n* = 15) were observed at work rate 1 (37.5 g GRS: *p* = 0.0679; 75 g GRS: *p* = 0.248), work rate 2 (37.5 g GRS: *p* = 0.723; 75 g GRS: *p* = 0.560), or work rate 3 (37.5 g GRS: *p* = 0.681; 75 g GRS: *p* = 0.351). Similarly, carbon dioxide production was not significantly altered in participants (*n* = 15) at work rate 1 (37.5 g GRS: *p* = 0.573; 75 g GRS: *p* = 0.0925), work rate 2 (37.5 g GRS: *p* = 0.834; 75 g GRS: *p* = 0.777), or work rate 3 (37.5 g GRS: *p* = 0.331; 75 g GRS: *p* = 0.445).

Additionally, respiratory rate in participants (*n* = 15) was higher at work rate 3 following the intake of 37.5 g GRS (*p* = 0.00660), but not 75 g GRS (*p* = 0.289; Figure S1). At work rate 1 and work rate 2, no differences in respiratory rate were observed between conditions (work rate 1: 37.5 g GRS, *p* = 0.682; 75 g GRS, *p* = 0.0394; work rate 2: 37.5 g GRS, *p* = 0.105; 75 g GRS, *p* = 0.449; Figure S1). No differences in ventilation were observed at any work rate (work rate 1: 37.5 g GRS, *p* = 0.248; 75 g GRS, *p* = 0.0539; work rate 2: 37.5 g GRS, *p* = 0.159; 75 g GRS, *p* = 0.741; work rate 3: 37.5 g GRS, *p* = 0.0693; 75 g GRS, *p* = 0.959; Figure S1). Furthermore, no differences in the rating of perceived exertion in participants (*n* = 15) were observed between conditions at any work rate among participants (RPE legs; work rate 1: 37.5 g GRS, *p* = 0.374; 75 g GRS, *p* = 0.388; work rate 2: 37.5 g GRS, *p* = 0.773; 75 g GRS, *p* = 0.111; work rate 3: 37.5 g GRS, *p* = 0.922; 75 g GRS, *p* = 0.895; RPE breathing; work rate 1: 37.5 g GRS, *p* = 0.876; 75 g GRS, *p* = 0.971; work rate 2: 37.5 g GRS, *p* = 0.281; 75 g GRS, *p* = 0.706; work rate 3: 37.5 g GRS, *p* = 0.452; 75 g GRS, *p* = 0.616; Figure S2).

### 3.6 Optimal dose modeling

To identify the dose of GRS that results in the lowest blood lactate concentration during submaximal exercise, we applied a quadratic modeling approach. This analysis utilized data from three administered doses (PLA, 37.5 g, and 75 g of GRS) in study participants (*n* = 15). The primary outcome variable was the change in blood lactate concentration at the highest submaximal work rate, using the PLA (0 g GRS) as a reference.

**FIGURE 6:**
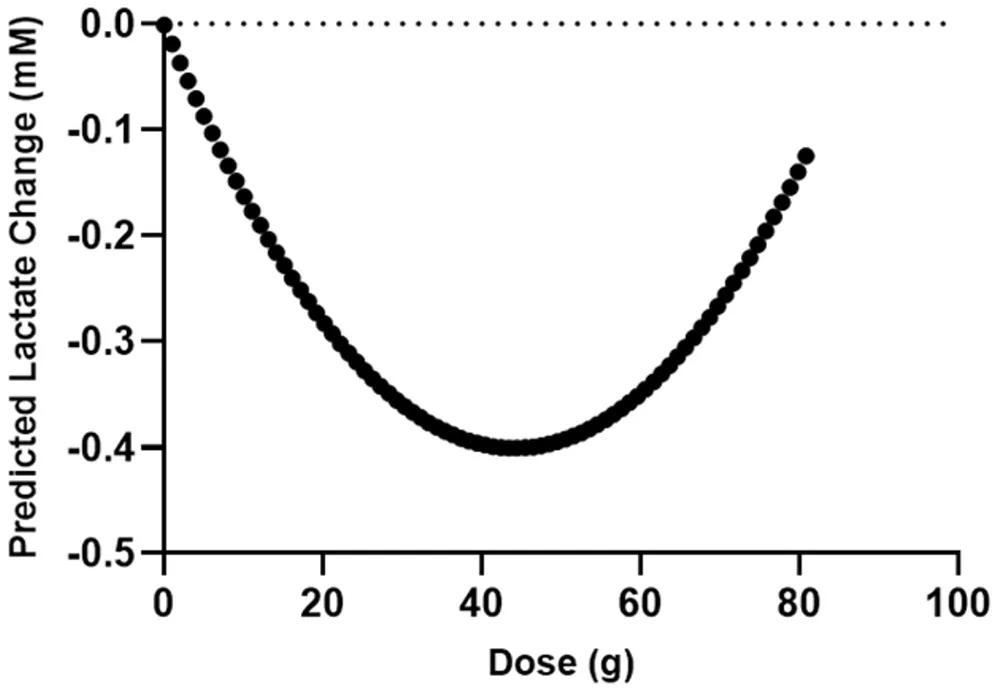
The quadratic regression model determined 44 g GRS as the optimal dose for reducing lactate accumulation in participants (*n* = 15; *R* ^2^ = 0.24; *p* = 0.0253).

We incorporated baseline lactate levels measured in the morning before dosing, as an additional variable to account for inter‐individual differences in lactate levels. A quadratic regression model was fitted to the data, and the optimal dose was determined by calculating the nadir of the quadratic curve. This calculation identifies the dose at which the dose–response curve achieves its minimum value, representing the most effective dose for reducing lactate accumulation. The optimal dose for minimizing lactate accumulation was calculated as 44 g of GRS. To statistically evaluate the dose–response relationship, we employed a linear mixed‐effects model. This model incorporated dose and baseline lactate levels as fixed effects and subject‐specific variability as random effects to account for individual differences. The coefficients for the quadratic terms had an *R* ^2^‐value of 0.24 and *p* = 0.0253, confirming a U‐shaped dose–response relationship (Figure 6).

## 4 DISCUSSION

The main finding of the present study was that acute intake of a GRS‐based beverage resulted in lower blood lactate concentrations at selected submaximal work rates corresponding to blood lactate concentrations of approximately 2.5–3.5 mM. In addition, blood glucose concentrations were altered at rest following beverage intake. Intriguingly, we observed a decrease in lactate levels with both the 37.5 g and 75 g GRScompared to PLA during cycling close to the participants' lactate threshold. This result is consistent with our previous findings from the one‐week GRS supplementation and HIIT‐training study. In that trial, the results could not be explained by differences in either VO_2_max or mitochondrial function or capacity (Flockhart et al., 2023), indicating other unknown mechanisms for changes in blood lactate and blood glucose metabolism.

Animal studies on isothiocyanate supplementation have investigated the mechanisms underlying alterations in lactate metabolism following isothiocyanate supplementation. Wang et al. reported that SFN pretreatment in mice increased the protein levels of MCT1 in the skeletal muscle during acute hypoxic exercise. Since MCT1 facilitates lactate transport across cell membranes in a bidirectional manner depending on concentration gradients and metabolic context, alterations in MCT1‐mediated transport may have contributed to the observed reduction in blood lactate levels. Additionally, the authors reported a significantly upregulated LDH activity which promoted the conversion of lactate to pyruvate (Wang et al., 2020). Overall, changes in the activity of the Cori cycle, which facilitates lactate clearance, represent an intriguing area for further investigation.

Clinical studies on dichloroacetate (DCA), a compound that stimulates pyruvate dehydrogenase (PDH), have demonstrated significantly reduced lactate levels during submaximal exercise and recovery, although not at maximal intensities. These findings suggest that limited PDH activity may play a role in the accumulation of plasma lactate during submaximal exercise in humans (Carraro et al., 1989). In the present study, the exercise intensity was moderate (RPE 10–15, Figure S2), and the modulation of PDH activity through isothiocyanate supplementation cannot be excluded as a contributing effect to the observed reductions in lactate levels. Supporting this notion, Plafker et al. (2025) demonstrated that SFN acutely reduces the inhibitory phosphorylation of pyruvate dehydrogenase, thereby promoting the entry of pyruvate into the citric acid cycle and reducing lactate production. Such a shift toward increased oxidative metabolism may have contributed to the observed reductions in blood lactate in the present study, although this mechanism remains speculative in the context of whole‐body exercise.

Blood glucose was significantly higher after both doses of GRS compared to PLA during rest and work rate 1. The increase in glucose levels could result from a lower oxidation of blood glucose or enhanced gluconeogenesis. However, during cycling at the lactate close to 4 mM, no significant differences in blood glucose were observed. Adding to these findings, we observed a small but significant increase in respiratory exchange ratio (RER) at the lowest work rate following the 75 g dose, coinciding with the highest blood glucose levels. At the highest work rate, there was a significant reduction in RER after the 37.5 g dose, which coincided with the lowest lactate concentrations. In our previous study, we found a tendency toward a lower RER after 1 week of supplementation (Flockhart et al., 2023). Moreover, studies have demonstrated that mitochondrial oxidation of long‐chain and short‐chain fatty acids is depressed in the absence of Nrf2 and accelerated when Nrf2 is constitutively active (Ludtmann et al., 2014). Further studies on humans are required to confirm if SFN and/or activation of Nrf2 induces changes in substrate preference.

A limitation of this study is the difference in carbohydrate content between the beverages. The placebo was isocaloric to the 37.5 g dose, but the 75 g dose contained double the amount of carbohydrates. However, the exercise tests were conducted 3 h after consumption, in the postprandial state. Furthermore, we did not observe any differences in glucose concentrations between the 37.5 and 75 g GRS doses, indicating that the difference in carbohydrate content was of minor importance. Another limitation is that we did not assess whether the lower lactate levels translated into improved performance. Theoretically, if the reduction in lactate results from the activation of pyruvate dehydrogenase, it could enhance performance by channeling more pyruvate into the mitochondria for efficient oxidation, reducing reliance on glycolysis, and thereby sparing muscle glycogen. Alternatively, if the lower lactate levels are due to increased activity of the hepatic Cori cycle, lactate could be more rapidly converted to glucose, possibly supporting glycogen resynthesis or maintaining blood glucose levels during exercise. Both mechanisms could potentially contribute to improved performance.

Isothiocyanates are electrophilic and have been shown to deplete endogenous glutathione (He et al., 2021) as well as induce single‐strand DNA breaks (Sestili et al., 2010). This mild stress is thought to enhance endogenous antioxidant systems over the long term, partly via the activation of Nrf2. Notably, we observed a reduction in myeloperoxidase levels approximately 3 h after GRS intake, suggesting a decrease in oxidative stress. This finding indicates that the adaptive compensatory system may act rapidly, likely within just a few hours of GRS consumption.

In conclusion, these findings suggest that acute intake of a glucosinolate‐rich beverage may influence lactate and glucose metabolism, with lower blood lactate concentrations observed at two submaximal work and altered blood glucose concentrations at rest.

## AUTHOR CONTRIBUTIONS

**Fredrik Vinge:** Data curation; formal analysis; investigation; methodology; visualization. **Emma Tillqvist:** Data curation; formal analysis; funding acquisition; investigation. **Oscar Horwath:** Data curation; formal analysis; investigation. **William Apró:** Data curation; formal analysis; investigation; methodology. **Filip J. Larsen:** Conceptualization; data curation; formal analysis; investigation; methodology; supervision; visualization. **Michaela L. Sundqvist:** Conceptualization;’data curation; formal analysis; funding acquisition; investigation; methodology; project administration; resources; supervision; visualization.

## FUNDING INFORMATION

This study was funded by grants from Dr. P Håkansson's foundation and Åke Wiberg's foundation (M24‐0086).

## CONFLICT OF INTEREST STATEMENT

Authors MLS and FJL hold a patent related to the use of isothiocyanates for enhancing adaptations to exercise training. In addition, they are co‐founders and shareholders in a commercial operational entity, which aims to market and sell products designed to augment athletic performance. The commercial operational entity had no role in the study design, data collection, data analysis, interpretation of the results, manuscript preparation, or the decision to publish. No other authors have any conflicts of interest to declare.

## ETHICS STATEMENT

This study has received ethical approval from the Swedish Ethical Review Authority (Nr: 2019‐02194).

## GENERATIVE AI DISCLOSURE STATEMENT

During the preparation and revision of this manuscript, the authors used Microsoft Words's text prediction features and Microsoft Copilot (Microsoft Corporation, 2025–2026) to assist with language editing, text refinement, and wording suggestions. The technology was used solely to improve clarity, readability, and the presentation of the text. AI assistance was used primarily in the Introduction and Discussion sections. It was not used for data analysis, interpretation of results, generation of scientific conclusions, or any other scientific decision‐making. All AI‐assisted content was reviewed, revised, and approved by the authors, who take full responsibility for the final content of the manuscript.

## Supporting information


**Figure S1.** Respiratory rate (a–c) and ventilation (d–f) during cycling at 1–3 work rates on an ergometer bike, 3 h after intake of a beverage containing 0, 37.5, or 75 g GRS. Data are presented as mean ± SD. Each dot represents one participant at the respective time point (*n* = 15). *p* < 0.05 was considered significant.
**Figure S2.** Rating of perceived exertion (RPE) in legs (a–c) and breathing (d–f) during cycling at 1–3 work rates on an ergometer bike, 3 h after intake of a beverage containing 0, 37.5, or 75 g GRS. Data are presented as mean ± SD. Each dot represents one participant at the respective time point (*n* = 15). *p* < 0.05 was considered significant.

## Data Availability

Data will be made available on request.
