# Supplementary material for: Effects of a glucosinolate‐rich beverage on blood lactate concentrations during submaximal exercise
Source: Physiol Rep. 2026 Aug 25;14(16):e71081. doi: 10.14814/phy2.71081 (PMC13507512; doi:10.14814/phy2.71081)
Supplement: Supplementary file 1 [file PHY2-14-e71081-s001.docx]

**SUPPLEMENTARY MATERIAL**

Supplementary material, Figure 1. Respiratory rate (a-c) and ventilation (d-f) during cycling at 1-3 work rates on an ergometer bike, 3h after intake of a beverage containing 0 g, 37.5 g, or 75 g GRS. Data are presented as mean ± SD. Each dot represents one participant at the respective time point (n = 15). p < 0.05 was considered significant.

Supplementary material, Figure 2. Rating of perceived exertion (RPE) in legs (a-c) and breathing (d-f) during cycling at 1-3 work rates on an ergometer bike, 3h after intake of a beverage containing 0 g, 37.5 g, or 75 g GRS. Data are presented as mean ± SD. Each dot represents one participant at the respective time point (n = 15). p < 0.05 was considered significant.

**QUANTIFICATION OF ISOTHIOCYANATES**

**Standards and Calibration Curve**

Glucosinolate and isothiocyanate standards (L-sulforaphane, glucoraphanin, glucoiberin and iberin) were obtained from Sigma-Aldrich (St. Louis, MO, USA). Stock solutions of each compound were prepared at a concentration of 1000 ng/μL and stored at -80ºC. An 11-point calibration curve (0.05-100 ng/µL) was prepared by serial dilutions and spiked with D6-salicylic acid as internal standards at a final concentration of 0.625 ng/μL.

**Sample Preparation**

Sample preparation of 100µl was performed according to A et al 2005, with some modification. In detail, 900 µL of extraction buffer (90/10 v/v methanol: water) including internal standards (13C9-phenylalanine, D4-cholic acid, 13C9-caffeic Acid and D6-salicylic acid) was added to each sample together with 1 tungsten bead. The sample was shaken at 30 Hz for 3 minutes in a mixer mill. The sample was centrifuged at +4 °C, 14 000 rpm (18 620g), for 10 minutes. 150 or 200 µl supernatant was transferred to micro vials for LC-MS analysis.

A small aliquot of the remaining supernatants was pooled and used to create quality control (QC) samples. MSMS analysis was run on the QC samples for identification purposes.

The samples were analyzed according to a randomized run order.

**LC-MS analysis**

Before LC-MS analysis the sample was re-suspended in 10 + 10 µL methanol and water. All samples were first analyzed in positive mode, then the instrument was switched to negative mode and a second injection of each sample was performed.

The chromatographic separation was performed on an Agilent 1290 Infinity UHPLC-system (Agilent Technologies, Waldbronn, Germany). 2 μL of each sample were injected onto an Acquity UPLC HSS T3, 2.1 x 50 mm, 1.8 μm C18 column in combination with a 2.1 mm x 5 mm, 1.8 μm VanGuard precolumn (Waters Corporation, Milford, MA, USA) held at 40 °C. The gradient elution buffers were A (H2O, 0.1 % formic acid) and B (75/25 acetonitrile:2-propanol, 0.1 % formic acid), and the flow-rate was 0.5 mL min-1. The compounds were eluted with a linear gradient consisting of 0.1 - 10 % B over 2 minutes, B was increased to 99 % over 5 minutes and held at 99 % for 2 minutes; B was decreased to 0.1 % for 0.3 minutes and the flow-rate was increased to 0.8 mL min-1 for 0.5 minutes; these conditions were held for 0.9 minutes, after which the flow-rate was reduced to 0.5 mL min-1 for 0.1 minutes before the next injection.

The compounds were detected with an Agilent 6546 Q-TOF mass spectrometer equipped with a jet stream electrospray ion source operating in positive or negative ion mode. The settings were kept identical between the modes, with exception of the capillary voltage. A reference interface was connected for accurate mass measurements; the reference ions purine (4 μM) and HP-0921 (Hexakis(1H, 1H, 3H-tetrafluoropropoxy)phosphazine) (1 μM) were infused directly into the MS at a flow rate of 0.05 mL min-1 for internal calibration, and the monitored ions were purine m/z 121.05 and m/z 119.03632; HP-0921 m/z 922.0098 and m/z 966.000725 for positive and negative mode respectively. The gas temperature was set to 150°C, the drying gas flow to 8 L min-1 and the nebulizer pressure 35 psig. The sheath gas temp was set to 350°C and the sheath gas flow 11 L min-1. The capillary voltage was set to 4000 V in positive ion mode, and to 4000 V in negative ion mode. The nozzle voltage was 300 V. The fragmentor voltage was 120 V, the skimmer 65 V and the OCT 1 RF Vpp 750 V. The collision energy was set to 0 V. The m/z range was 70 - 1700, and data was collected in centroid mode with an acquisition rate of 4 scans s-1 (1977 transients/spectrum).

**Data analysis-evaluation/statistical methods**

All data processing was performed using the Agilent Masshunter Profinder version B.10.0.2 (Agilent Technologies Inc., Santa Clara, CA, USA) using the Batch Targeted feature extraction in Masshunter Profinder and the glucosinolates as target library. The identification of the metabolites was based on MS, MSMS and retention time information. The concentration of each glucosinolate was calculated based on the calibration curves.
